# A Diagnostic Conundrum: A Case of Pediatric Autoimmune Pancreatitis

**DOI:** 10.1097/PG9.0000000000000326

**Published:** 2023-06-09

**Authors:** Naseem Ravanbakhsh, Nick Shillingford, Travis L. Piester

**Affiliations:** From the * Division of Gastroenterology, Hepatology and Nutrition, Children’s Hospital Los Angeles, Los Angeles, CA, USA; † Division of Pathology, Children’s Hospital Los Angeles, Los Angeles, CA, USA.

**Keywords:** autoimmune pancreatitis (AIP), obstructive jaundice, abdominal pain, ERCP, endoscopic ultrasound, fine needle biopsy, malignancy

## Abstract

Autoimmune pancreatitis (AIP) is rare cause of abdominal pain in children who often present with obstructive jaundice, mimicking malignancy. An investigation of clinical symptoms, serology, imaging, and histopathology is necessary for diagnosis. We report a 10-year-old female presenting with abdominal pain and jaundice, ultimately found to have AIP after confirmation with tissue pathology. Our patient’s prompt response to corticosteroid initiation is characteristic of this disease state. AIP has 2 subtypes, the second of which is more frequently found in children. Our patient’s pathology did not fit perfectly with either subtype, but had features found in each one. While diagnostic criteria for AIP have not established in pediatrics, our case highlights the combination of clinical symptoms, imaging, and histopathology that children classically present with. While rare, the diagnosis of AIP is associated with comorbidities and must be considered in any child presenting with a pancreatic mass or biliary stricture.

**Clinical Pearls**

- Autoimmune pancreatitis (AIP) is a rare disease that can mimic multiple disease processes, including neoplastic disorders (1).
- Characterized by obstructive jaundice, abdominal pain, and prompt response to corticosteroids, AIP continues to be a complex disease that requires a thorough investigation of clinical symptoms, serology, imaging, and histopathology for accurate diagnosis (2).

## INTRODUCTION

Autoimmune pancreatitis (AIP) is a rare disease that can mimic multiple disease processes, including neoplastic disorders (1). Characterized by obstructive jaundice, abdominal pain, and prompt response to corticosteroids (2), AIP continues to be a complex disease that requires a thorough investigation of clinical symptoms, serology, imaging, and histopathology for accurate diagnosis.

## CASE REPORT

**FIGURE 1.**
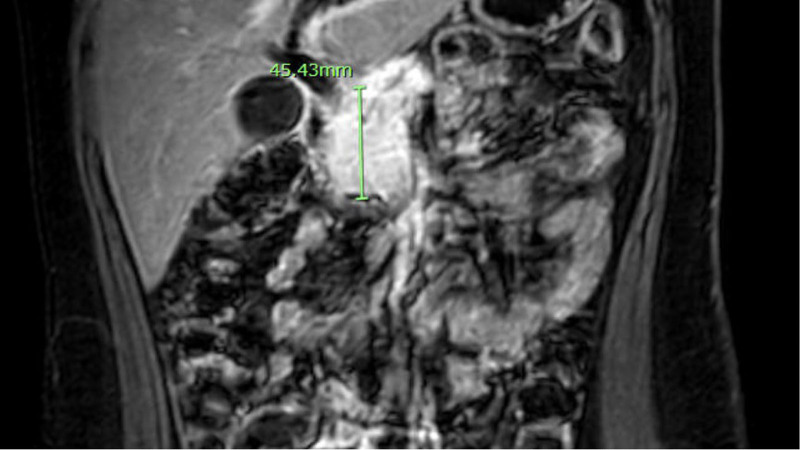
Obstructing mass involving the pancreatic head and uncinate process.

**FIGURE 2.**
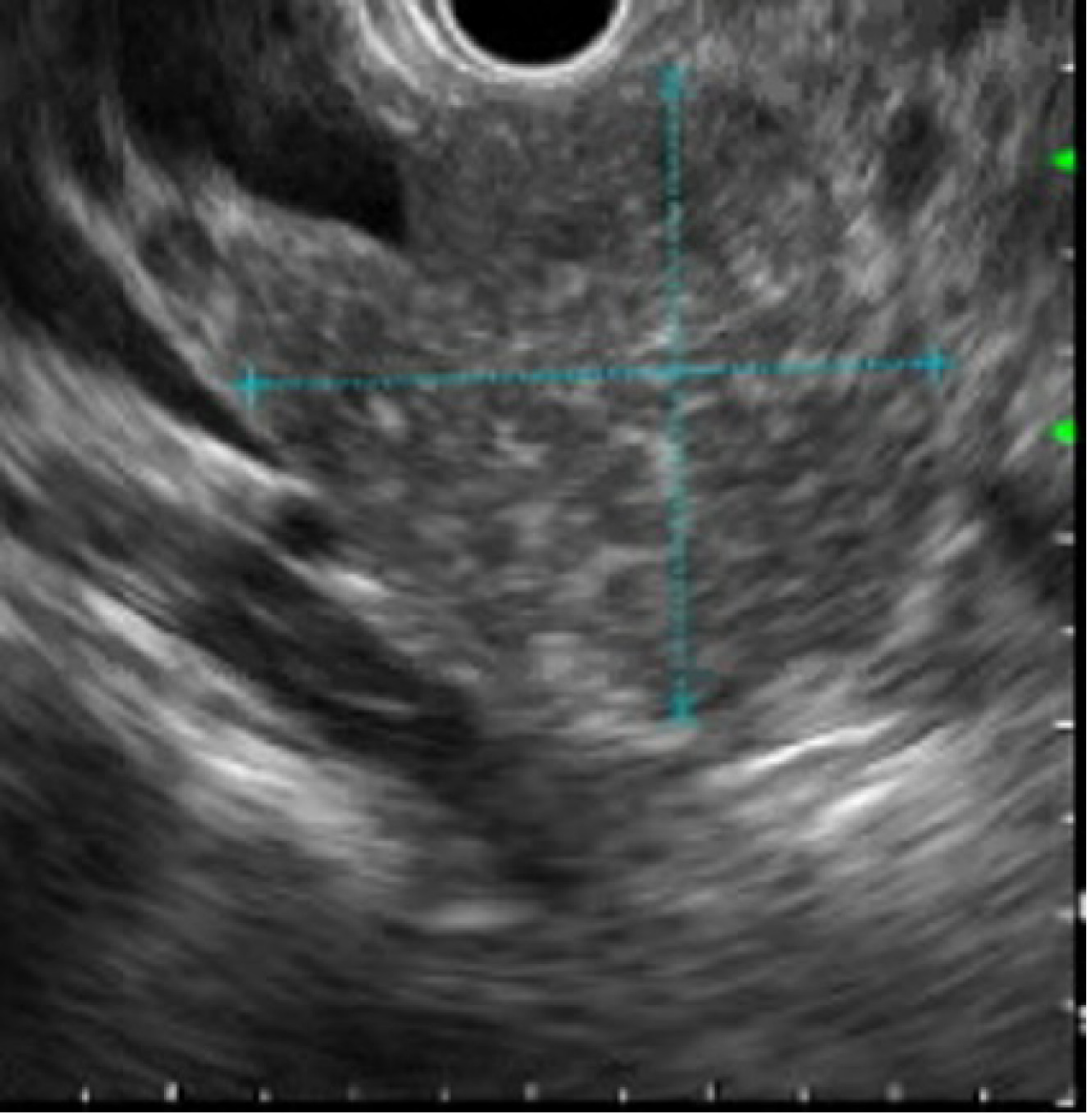
Endoscopic ultrasound findings significant for a heterogenous pancreatic parenchyma without a definite mass.

**FIGURE 3.**
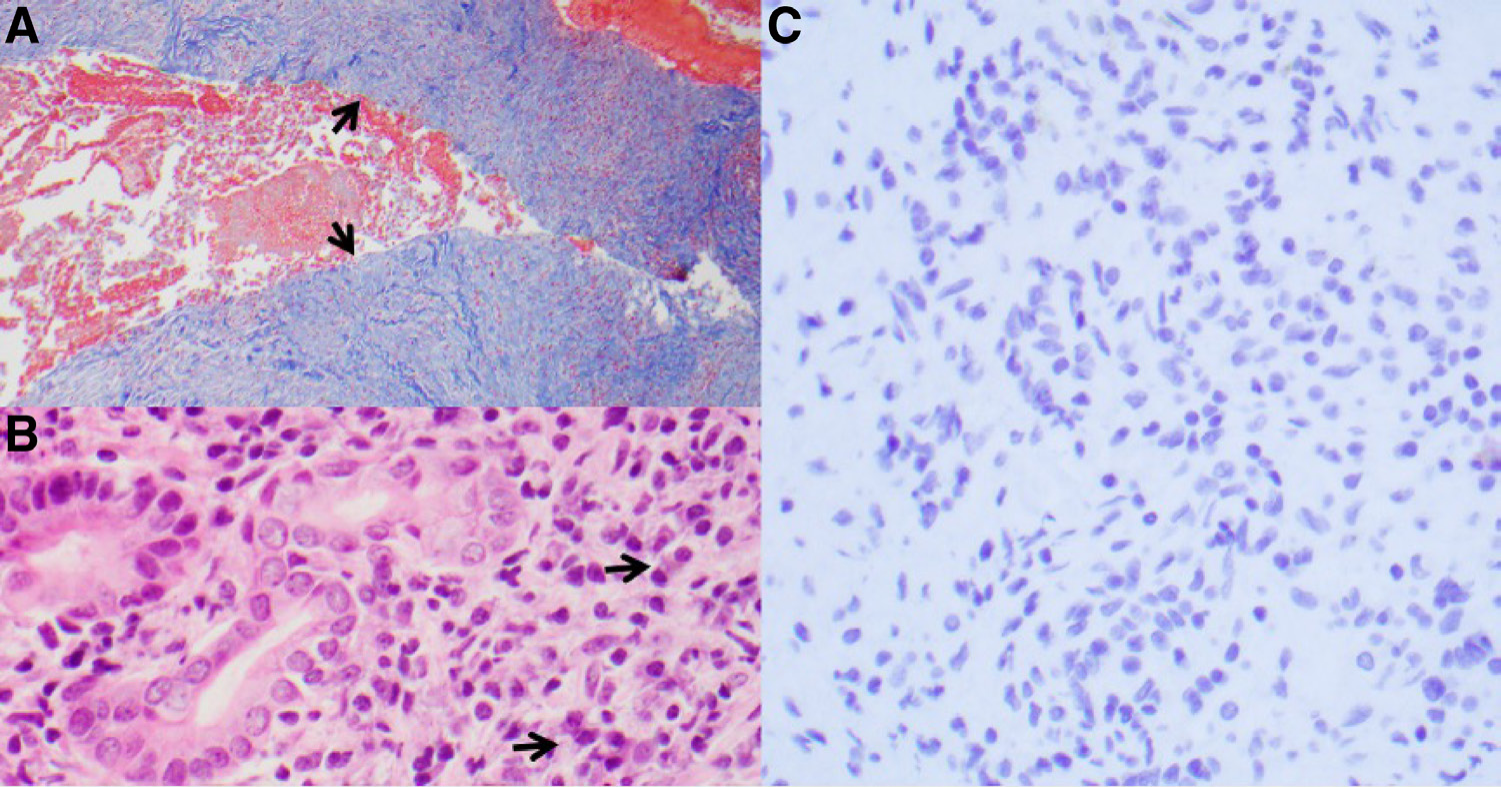
Histopathology findings. A) Masson trichrome stain highlights fibrotic nature of tissue cores. B) Chronic inflammatory infiltrate composed of lymphocytes and plasma cells. C) IgG4-negative plasma cells.

Ten-year-old previously healthy female presented with several months of abdominal pain and 4 days of worsening jaundice and scleral icterus. Initial labs were significant for elevated transaminases: alanine transaminase of 230 units/L, aspartate transaminase of 133 units/L, conjugated bilirubinemia: total bilirubin of 5.04 mg/dL with a conjugated component of 1.65 mg/dL, alkaline phosphatase of 512 units/L, and gamma-glutamyl transpeptidase of 440 units/L. nitial abdominal ultrasound showed a heterogeneous, hypoechoic mass in the region of the pancreatic head, common bile duct (CBD) dilation to 10 mm, and intrahepatic duct dilation. Magnetic resonance cholangiopancreatography demonstrated the obstructing mass involving the pancreatic head and uncinate process, causing extrahepatic and intrahepatic biliary duct dilation as well as pancreatic duct dilation to 3 mm (Fig. 1). Imaging was also concerning for a lesion within the right hepatic lobe. Given concern for malignancy with possible hepatic metastasis, oncology was consulted: tumor markers were significant for an elevated cancer antigen 19-9 of 41 units/mL (reference range: <34 units/mL), alpha-fetoprotein of 1.6 ng/mL (reference range: 0.8–7.3 ng/mL), and a carcinoembryonic antigen <0.5 ng/mL (reference range: <2.5 ng/mL). The patient underwent endoscopic retrograde cholangiopancreatography (ERCP) with CBD stenting, endoscopic ultrasound (Fig. 2), and fine needle biopsy of the pancreatic head. Histology showed extensive fibrosis, brisk chronic inflammation, scattered plasma cells, obliterative phlebitis, and parenchymal atrophy with extensive parenchymal loss (Fig. 3). There was no evidence of malignancy. Findings were overall consistent with AIP. The patient was started on corticosteroids at 1 mg/kg/d.

Within 6 days, the patient’s symptoms, stricture, and labs improved with placement of the CBD stent and steroid therapy. The patient developed hyperglycemia before steroid initiation and was diagnosed with new-onset diabetes necessitating Metformin. The patient underwent a repeat magnetic resonance imaging abdomen days later to evaluate the hepatic lesion, which was found to be most compatible with a hemangioma. Repeat magnetic resonance imaging 1 month later showed improvement in the pancreatic head mass without biliary stricture. The patient completed a course of corticosteroids at 60 mg for 1 month followed by a taper over 10 weeks. Once the steroid course was completed, the Metformin was weaned too, as glucose levels had normalized. The patient underwent several ERCPs after hospital discharge over several months, at which time the distal CBD stricture was dilated, and stents were changed. The stent was ultimately removed about 8 months from initial presentation and ERCP. One year after hospitalization, the patient was clinically asymptomatic and off all medications.

## DISCUSSION

AIP is a distinct form of pancreatitis, characterized by abdominal pain and/or obstructive jaundice with or without a pancreatic mass (2). Subtypes include lymphoplasmacytic sclerosis pancreatitis (type 1) and idiopathic duct-centric pancreatitis (type 2). Type 1 AIP is more prevalent in Asia, mainly described in adults, and is associated with elevated serum immunoglobulin G4 (IgG4) (3). Type 2 AIP is non-IgG4-related, prevalent in the Europe and the United States (3), and more frequently found in children (2,4). Histology varies between the 2 types of AIP: type 1 AIP is commonly associated with lymphoplasmacytic sclerosing pancreatitis with storiform fibrosis, obliterative phlebitis, and IgG4+ plasma cells, while type 2 AIP is characterized by idiopathic duct-centric pancreatitis with granulocytic epithelial lesions, sometimes including lobular neutrophilic infiltration (4). Clinical response to steroids is seen in both types of AIP (1–6,7).

Our patient exhibited histopathology features from both subtypes of AIP: pancreatic pathology demonstrated lymphoplasmacytic infiltrates with extensive fibrosis and brisk chronic inflammation with scattered plasma cells and obliterative phlebitis, features seen in type 1 AIP. Increased serum IgG4 and IgG4 positive plasma cell density was not seen. Pathology also demonstrated lymphoplasmacytic infiltrates, which is seen in type 2 AIP. Often, histological features of pediatric AIP include a combination of granulocytic epithelial lesions, lymphoplasmacytic infiltration, and fibrosis. While it is possible that pediatric AIP may have its own distinct histology, such findings have not been validated (2,5).

Diagnostic criteria for AIP have been established in the adult population through the histology, imaging, serology, other organ involvement, and response to therapy (HISORt) criteria (6). Diagnostic criteria in children are not as clear but can be recognized from a combination of clinical symptoms and specific imaging findings. Children commonly present with abdominal pain and/or obstructive jaundice in combination with imaging findings of focal pancreas enlargement, main pancreatic duct irregularities, and distal CBD narrowing/stricture (5).

Complications of AIP in children include recurrent disease (found in 17% of the patient population in Scheers et al study), exocrine pancreatic insufficiency, and diabetes mellitus. Studies of children and adults with AIP have found a subset of patients with AIP who also have autoimmune diseases like Inflammatory Bowel Disease, though it is unclear which disease state precipitates the other (5).

Corticosteroids are the treatment for AIP. In children, an induction dose of 1–1.5 mg/kg per day is utilized, which has been shown to improve acute symptoms in most cases. Steroids may also help prevent long-term complications of AIP like pancreatic insufficiency, although more studies are needed to help elucidate this potential. Repeat imaging at 3 months after initiation of steroids may be useful in evaluating for normalization of the pancreas and confirming the diagnosis of AIP (2).

## CONCLUSION

While rare, the diagnosis of AIP in children is associated with multiple comorbidities and must be considered in any child presenting with pancreatic mass or biliary stricture. Histologic confirmation of AIP with fine needle biopsy may be warranted and should be considered to help confirm the diagnosis, differentiate from malignancy versus other etiologies, guide treatment, and avoid surgery (8,9,10). Prompt treatment with steroids has shown excellent short-term benefit and may help prevent long-term complications (2).

## ACKNOWLEDGMENTS

Both verbal and written consent from the parent was obtained for publication of the case.
